# Direct nitrogen, phosphorus and carbon exchanges between Mucoromycotina ‘fine root endophyte’ fungi and a flowering plant in novel monoxenic cultures

**DOI:** 10.1111/nph.18630

**Published:** 2023-02-05

**Authors:** Grace A. Hoysted, Katie J. Field, Besiana Sinanaj, Christopher A. Bell, Martin I. Bidartondo, Silvia Pressel

**Affiliations:** ^1^ Plants, Photosynthesis and Soil, School of Bioscience University of Sheffield Sheffield S10 2TN UK; ^2^ Centre for Plant Sciences University of Leeds Leeds LS2 9JT UK; ^3^ Department of Life Sciences Imperial College London London SW7 2AZ UK; ^4^ Department of Ecosystem Stewardship Royal Botanic Gardens, Kew Richmond TW9 3DS UK; ^5^ Department of Life Sciences Natural History Museum London SW7 5BD UK

**Keywords:** arbuscular mycorrhizal fungi, carbon, clover, fine root endophytes, flowering plants, Mucoromycotina, soil nutrients, symbiosis

## Abstract

- Most plants form mycorrhizal associations with mutualistic soil fungi. Through these partnerships, resources are exchanged including photosynthetically fixed carbon for fungal‐acquired nutrients. Recently, it was shown that the diversity of associated fungi is greater than previously assumed, extending to Mucoromycotina fungi. These Mucoromycotina ‘fine root endophytes’ (MFRE) are widespread and generally co‐colonise plant roots together with Glomeromycotina ‘coarse’ arbuscular mycorrhizal fungi (AMF). Until now, this co‐occurrence has hindered the determination of the direct function of MFRE symbiosis.
- To overcome this major barrier, we developed new techniques for fungal isolation and culture and established the first monoxenic *in vitro* cultures of MFRE colonising a flowering plant, clover. Using radio‐ and stable‐isotope tracers in these *in vitro* systems, we measured the transfer of ^33^P, ^15^N and ^14^C between MFRE hyphae and the host plant.
- Our results provide the first unequivocal evidence that MFRE fungi are nutritional mutualists with a flowering plant by showing that clover gained both ^15^N and ^33^P tracers directly from fungus in exchange for plant‐fixed C in the absence of other micro‐organisms.
- Our findings and methods pave the way for a new era in mycorrhizal research, firmly establishing MFRE as both mycorrhizal and functionally important in terrestrial ecosystems.

## Introduction

Among Earth's most important symbioses are the ancient plant–fungus partnerships known as mycorrhizas, or ‘mycorrhiza‐like’ associations in plants without roots (Read *et al*., 2000). These mutualisms, underpinned by the bidirectional exchange of plant‐fixed carbon for fungal‐acquired mineral nutrients (Raven & Allen, 2003), were instrumental in plant terrestrialisation > 500 million years ago (Morris *et al*., 2018) by facilitating early, rootless plant access to mineral nutrients held within primeval soils (Pirozynski & Malloch, 1975; Field *et al*., 2015a). Thus, fungi played a formative role in the development of Earth's terrestrial ecosystems and climate through their contributions to global carbon and nutrient cycles (Taylor *et al*., 2011; Mills *et al*., 2018). Today, these associations are formed between most land plants and a diverse subset of soil fungi (Field & Pressel, 2018), including the widespread arbuscular mycorrhizal fungi (AMF) in the subphylum Glomeromycotina (Brundrett & Tedersoo, 2018), estimated to occur in > 70% of plants (Smith & Read, 2008).

Until recently, AMF encompassed the globally distributed ‘fine root endophytes’ (FRE; *Glomus tenue*; Thippayrugs *et al*., 1999), which are known to colonise several vascular plant families (Ali, 1969; Abbott, 1982; Thippayrugs *et al*., 1999), but have been largely overlooked due to practical limitations in molecular detection and inability to study them apart from coexisting AMF (Orchard *et al*., 2017a). Through improved molecular detection and identification (Bidartondo *et al*., 2011), it is now clear that FRE are distinct from Glomeromycotina AMF, belonging instead to the Endogonales in the subphylum Mucoromycotina (Orchard *et al*., 2017a), and recently renamed *Planticonsortium tenue* (Walker *et al*., 2018). Thus, FRE previously reported in flowering plants (Ali, 1969; Abbott, 1982; Thippayrugs *et al*., 1999) are likely closely related to Endogonales fungal associates previously identified (Bidartondo *et al*., 2011; Rimington *et al*., 2015; Hoysted *et al*., 2018, 2019) and shown to be mutualistic in terms of carbon‐for‐nutrient exchange in a range of nonflowering plants, albeit using nonsterile, soil‐based experimental systems (Field *et al*., 2015b, 2016, 2019; Hoysted *et al*., 2019, 2020).

Colonisation by FRE is, like AMF, generally characterised by the presence of arbuscules and arbuscule‐like structures (Orchard *et al*., 2017b), while the small diameter of FRE hyphae (< 1.5 μm), with small swellings and ‘fan‐like’ morphologies, is considered a distinctive trait that separates them from AMF (or ‘coarse root endophytes’) which consistently develop wider (> 3 μm in diameter) hyphae and larger vesicles (Orchard *et al*., 2017a). Morphological plasticity has been noted in transmission and scanning electron micrographs of the ultrastructure of Mucoromycotina FRE (MFRE) exclusively associating with liverworts (Field *et al*., 2015b, 2016) and a vascular plant (Hoysted *et al*., 2019). Most recently, cryo‐SEM has confirmed uniformly thin hyphae and hyphal ‘ropes’ as potential diagnostic features of MFRE symbioses (Albornoz *et al*., 2020). Differently from the strictly biotrophic AMF, MFRE are considered facultative saprotrophs as it has been possible to isolate them from host plants – both a nonvascular (Field *et al*., 2015b) and vascular plant (as shown herein) – and to grow them axenically, that is without a host in culture.

Latest research indicates that MFRE play a nutritionally complementary role to AMF by facilitating plant nitrogen (N) assimilation alongside AMF‐facilitated plant phosphorus (P) acquisition through co‐colonisation of the same host (Field *et al*., 2019). Such functional complementarity is further supported by the observation that MFRE transfer significant amounts of ^15^N but relatively little ^33^P isotope tracers to a host lycophyte in the first experimental demonstration of MFRE nutritional mutualism with a vascular plant (Hoysted *et al*., 2019). The apparent ability of MFRE, but not AMF, to transfer N also from organic sources to host liverworts in nonsterile soil (Field *et al*., 2019) together with their presumed facultative saprotrophic nature points to possible functional similarities with ectomycorrhizal fungi, an assumption in line with results from a recent network analysis of symbiotic fungal associations in liverworts (Rimington *et al*., 2019). However, evidence for the precise role of MFRE, in the absence of other soil micro‐organisms, remains equivocal.

To date, detailed research into plant–MFRE associations has been constrained by a lack of *in vitro* experimental systems that allow indisputable determination of the direct function of the MFRE symbiosis in isolation. Our recent knowledge of MFRE function has been derived largely from experiments using wild soil‐based systems and wild‐collected plants that naturally only associate with MFRE (Field *et al*., 2015b, 2016, 2019; Hoysted *et al*., 2019, 2020), or from soil culture‐based experimental pots (Orchard *et al*., 2017a; Albornoz *et al*., 2020). Each of these methods has significantly enhanced our understanding of MFRE form and function, shedding new light on the importance of MFRE associations in nature, and remains useful in the studies of plants associating with mixed microbial communities.

However, the development of *in vitro* experimental systems capable of distinguishing between fungal symbionts in the absence of other soil biota is now critical for the functional significance of MFRE associations to be fully defined (Sinanaj *et al*., 2021). This is particularly important as evidence is increasingly pointing towards most plants forming simultaneous symbioses with AMF and MFRE (Field *et al*., 2015a,b, 2016; Hoysted *et al*., 2019, 2020) and there being complementarity in function between symbionts (Field *et al*., 2019; Hoysted *et al*., 2019). Recently, it was reported that a free‐living Mucoromycotina, *Gongronella* sp. W5, utilises plant sucrose as a carbon source (Wang *et al*., 2021); however, data showing mutualistic transfer of carbon‐for‐nutrients between MFRE and a plant host in the absence of other micro‐organisms do not currently exist. The development of an *in vitro* experimental system is critical to achieve this and further understand function, development and signalling and to identify specific symbiotic structures and interfaces in MFRE, particularly for comparisons with model AM symbioses. Here, we resolve this research challenge by establishing experimentally tractable, monoxenic symbiotic cultures of MFRE and white clover (*Trifolium repens*), a flowering plant genus used in other recent studies of MFRE colonisation (e.g. Orchard *et al*., 2017a; Albornoz *et al*., 2020), albeit in nonsterile systems. Using radio‐ and stable‐isotope tracers in our *in vitro* systems, we measured the transfer of ^33^P, ^15^N and ^14^C between MFRE hyphae and the host plant to provide the first unequivocal evidence of mutualistic transfer of MFRE‐assimilated nutrients for plant‐fixed carbon with a flowering plant, in the absence of other microbes.

## Materials and Methods

### Isolation of MFRE symbionts

*Lycopodiella inundata* (L.) Holub gametophytes and young sporophytes (with protocorms; Hoysted *et al*., 2019) were collected from Thursley Nature Reserve, Surrey, UK (SE 90081 39754) in September 2019 and processed immediately for fungal isolation or stored in their natural substrate in growth chambers at 20°C : 15°C day : night temperatures and a 16‐h day length, 225 μmol photons m^−2^ s^−1^, for molecular analyses.

**Fig. 1:**
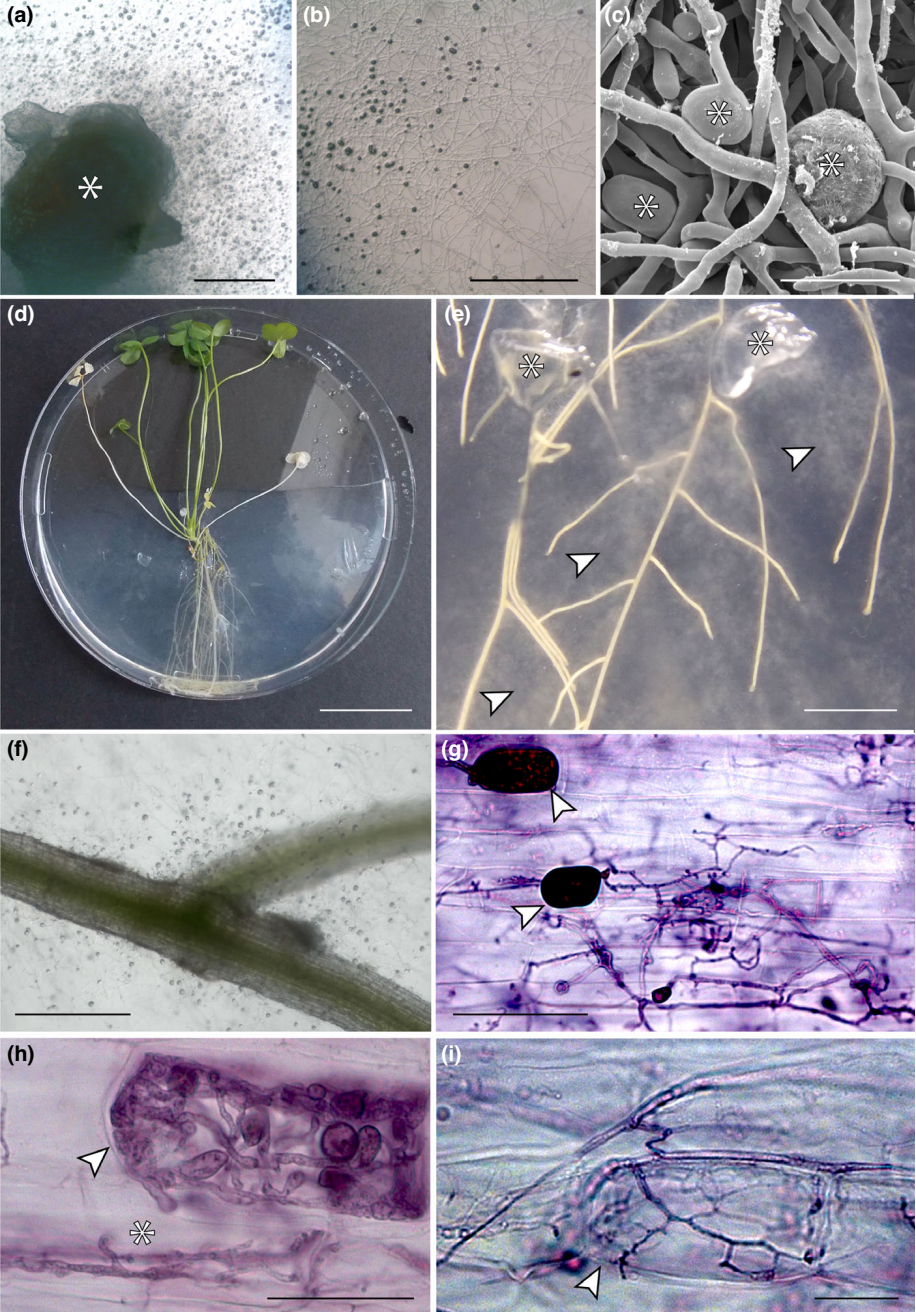
*In vitro* isolation of Mucoromycotina fine root endophytes (MFRE) from *Lycopodiella inundata* (a–c) and colonisation of white clover (*Trifolium repens*) by isolated MFRE (d–i). (a) *L. inundata* gametophyte (*) with copious fungal outgrowth, magnified in (b) and imaged under a scanning electron microscope (c); note the fine hyphae with numerous swellings (*). (d, e) Monoxenic culture of *T. repens* and isolated MFRE after 12 wk of culture; (e) note the abundant mycelium extending from plugs of pure MFRE cultures (*) and enveloping the roots (arrowed), enlarged in (f) (see also Fig. 4). (g–i) Trypan blue/ink‐stained roots of *Trifolium* showing fine, irregularly branching hyphae with larger vesicles or spores (g, arrowed), forming tightly wound intracellular coils with small intercalary and terminal swellings (h, arrowed); note also the subtending intracellular hyphal ropes (*). (i) Young, arbuscule‐like structure (arrowed) forming inside a clover root cell. Bars: (d) 50 mm; (e) 2 mm; (a) 500 μm; (b, f) 300 μm; (g) 50 μm; (c, h, i) 20 μm.

Gametophytes and young sporophytes were carefully cleaned of adhering substratum; rhizoids and (for sporophytes) leaves were removed before thorough rinsing in dH_2_O by gentle shaking for 1 h, followed by surface‐sterilisation for 1 min in 0.5% sodium hypochlorite. Sterile gametophytes were kept intact or halved, while the sporophytic protocorms were cut into *c*. 0.5‐mm sections, placed onto fungal growth medium under sterile conditions and incubated in the dark at 27°C. The fungal medium was the same as that developed by Field *et al*. (2015b) except for a lower concentration of thiamine (thiamine HCl, 100 μg). Once fungal outgrowth from plant fragments became visible (1–2 wk), hyphae were subcultured onto the same medium used for isolation and kept in the dark at 27°C (Fig. 1a–c).

### Molecular identification of fungal symbionts

Molecular analyses of fungal symbionts of *Lycopodiella* were carried out within 1 wk of collection (Rimington *et al*., 2015). Extraction and sequencing of DNA were performed using the method of Bidartondo *et al*. (2011). The universal fungal 18S primer combination NS1 (White *et al*., 1990) and EF3 (Smit *et al*., 1999) was used to amplify DNA which was cloned (Topota; Invitrogen) and sequenced using an Applied Biosystems Genetic Analyser 3730 (Waltham, MA, USA). Between four and eight clones were sequenced for each of eight samples and identified using NCBI Blast (Altschul *et al*., 1997). Sequence editing and assembly were performed in Geneious v.5.6 (http://geneious.com). The alignment algorithms of Muscle were used within Mega v.5.1 (Tamura *et al*., 2013), with reference sequences from GenBank. Using Uchime (Edgar *et al*., 2011) within Mothur (http://www.mothur.org), confirmed sequences were not chimeric. Evolutionary models were tested in Mega. Bayesian inference was carried out using MrBayes (Huelsenbeck & Ronquist, 2001) and FigTree v.1.4 (http://tree.bio.ed.ac.uk) for visualisation and editing. The same method was used for molecular identification of the fungus isolated from *Lycopodiella* and introduced in monoxenic microcosms with clover, as well as that of the fungus colonising the roots of clover in our monoxenic microcosms.

### Axenic plants

Clover plants were cultured *in vitro* to establish monoxenic cultures with MFRE isolates. Seeds were surface‐sterilised in 70% ethanol for 1 min, rinsed in water, followed by shaking in 5% commercial sodium hypochlorite for 30 min and then thorough rinsing in sterilised dH_2_O. Seeds were placed in 140‐mm triple‐vented sterile Petri dishes containing modified Strullu–Romand (MSR) medium lacking vitamins and sucrose (Declerck *et al*., 1998) solidified with 0.4% Phytagel (Sigma‐Aldrich), and adjusted to pH 5.5 before sterilisation, where they germinated in the dark, inverted at 27°C, after 3 d.

### *In vitro* colonisation of clover by MFRE isolate

**Fig. 2:**
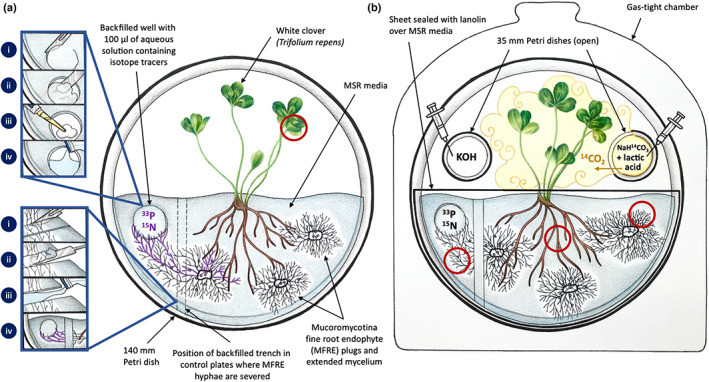
Set‐up of *in vitro* monoxenic experimental system used to quantify fluxes of nutrients exchanged between Mucoromycotina fine root endophytes (MFRE) and white clover (*Trifolium repens*). (a) Monoxenic cultures were developed in sterile conditions in 140 mm Petri dishes half‐filled with modified Strullu–Romand (MSR) media, which lacked vitamins and sucrose, solidified with 0.4% Phytagel and poured at a slant. Three plugs of axenic MFRE culture were placed on the media and allowed to establish before the introduction of a *T. repens* seedling. Plates were sealed for 12 wk. One hundred microlitres of an aqueous solution containing ^33^P or ^15^N was added to a well in each plate (see top‐left detail panel). In the control plates, a trench was cut and subsequently filled with sterile medium to sever MFRE and to ensure there was no direct fungal access to radio‐ and stable‐isotope tracers (see bottom‐left detail panel). Purple colour on hyphae denotes the flow of fungal‐acquired isotope tracers. (b) The MSR media and its contents were sealed off from the aboveground plant tissue, and each plate was placed in a gas‐tight chamber before ^14^C‐labelling. Lactic acid was added to ^14^C‐labelled sodium bicarbonate to release a 0.5 Mbq pulse of ^14^CO_2_ for the plant to fix. At the end of the 24‐h labelling period, KOH was added to the system to capture any remaining headspace ^14^CO_2_. Red circles in (a, b) denote regions that were sampled for the analysis of ^33^P, ^15^N and ^14^C.

The same MSR medium used for seed germination was plated slanted in 140 mm sterile, triple‐vented raised Petri dishes. Three‐day‐old axenic clover seedlings (one per dish) were placed with their root system adhering to the medium (and the shoot extending into the medium‐free portion of the dish) in Petri dishes pre‐inoculated (1 wk) with axenic MFRE hyphae. Control plants were placed in noninoculated dishes containing the same medium. The sterile monoxenic microcosms were sealed with Parafilm, and the root system was covered with aluminium foil to prevent photo‐oxidation and placed vertically in a growth chamber at 27°C, with a 16‐h photoperiod and a photosynthetic photon flux of 300 μmol m^−2^ s^−1^. Plates were undisturbed for 12 wk to allow time for the fungus to colonise plant roots. After 12 wk, fungal hyphae were growing in close association with clover roots (Figs 1d–f, 2a); roots were therefore harvested, cleared in 10% KOH and either stained with trypan blue (Hoysted *et al*., 2019) or 5% ink‐vinegar (Vierheilig *et al*., 1998).

### Quantification of fluxes of ^33^P, ^15^N and ^14^C between MFRE and clover

*In vitro* clover cultures were split into three groups: monoxenic plates colonised by MFRE with hyphae intact and, as controls, monoxenic plates colonised by MFRE with hyphae severed and plates containing only axenic clover without fungus. One hundred microlitres of aqueous solution containing 0.5 MBq ^33^P‐orthophosphate (111 TBq mmol^−1^ SA, 0.15 ng ^33^P supplied; Hartmann Analytics) and ^15^N‐ammonium chloride (1 mg ml^−1^; 0.1 mg ^15^N added; Sigma‐Aldrich) was introduced into a well in each of the plates (Fig. 2a, top‐left detail panel). In the plates of clover colonised by MFRE that served as controls, a trench was cut in the medium using a sterile blade and subsequently backfilled with sterile medium to sever hyphae and prevent any direct fungal access to radio‐ and stable‐isotope tracers (Fig. 2a, bottom‐left detail panel). As a further control, we included isotope‐containing wells in microcosms containing only clover to observe the direct isotope tracer uptake of the asymbiotic plant roots compared with plants that formed mycorrhizal associations with the MFRE isolate. By backfilling trenches with additional medium, diffusion of isotopes from the well was purposefully permitted in all microcosms, allowing us to account for the role of intact MFRE hyphae.

The plates were resealed with Parafilm in sterile conditions and placed in controlled environment chambers (Model no. MicroClima 1200; Sneijders Labs, Tilburg, the Netherlands) with 16‐h daytime (20°C and 70% humidity) and 8‐h night‐time (15°C and 70% humidity). Daytime photosynthetically active radiation (PAR), supplied by LED lighting, was 225 μmol photons m^−2^ s^−1^. Atmospheric [CO_2_] was set at 440 μl l^−1^, monitored using a sensor system (Vaisala, Birmingham, UK) and maintained through the addition of gaseous CO_2_. Assimilation of ^33^P into the aboveground plant material was monitored using a hand‐held Geiger counter held over the Petri dish.

For ^14^C‐labelling, the medium containing clover roots and MFRE hyphae was sealed off from the aboveground plant tissue and each plate placed in a gas‐tight chamber (Fig. 2b). A 0.5‐MBq pulse of ^14^CO_2_ was then liberated into the headspace of sealed Petri dishes by adding 2 ml of 90% (w/v) lactic acid to 13.5 μl NaH^14^CO_3_ (specific activity 2.124 GBq mmol^−1^; Hartmann Analytics; Fig. 2b). Cultures inside sealed chambers were maintained under growth conditions as previously mentioned. Twenty‐four hours after the addition of ^33^P and ^15^N tracers and liberating the pulse of ^14^CO_2_, the plates were opened and root‐and‐shoot material was separated, freeze‐dried, weighted and homogenised. For the analysis of ^33^P, between 0.1 and 4 mg of shoot, root and fungal material was digested in 500 μl of concentrated sulphuric acid at 365°C for 15 min. Fifty microlitres of hydrogen peroxide was added to cooled samples and returned to the digest block (BT5D; Grant Instruments, Cambridge, UK). Cleared digest solutions were then diluted to 5 ml with dH_2_O. ^33^P‐radioactivity of plant material was quantified through liquid scintillation (Tri‐Carb® 3100TR; PerkinElmer, Beaconsfield, UK). One millilitre of each digest solution was added to 10 ml of Elmusify‐safe scintillant, and ^33^P content was calculated using Eqn 1.

(Eqn 1) $$M^{33}P=\left\{\left[\frac{\frac{\text{cDPM}}{60}}{\text{SAct}}\right]M_{\mathrm{wt}}\right\}\mathrm{Df}$$

where *M* ^33^P, mass of ^33^P (mg); cDPM, counts as disintegrations per min; SAct, specific activity of the course (Bq mmol^−1^); Df, dilution factor; and *M*_wt_, molecular mass of P (Cameron *et al*., 2007).

Between 0.1 and 4 mg of freeze‐dried, homogenised root‐and‐shoot tissues were weighed into 6 × 4 mm^2^ tin capsules (Sercon, Crewe, UK), and ^15^N abundance was determined using a continuous flow infrared mass spectrometry (IRMS; model no. PDZ 2020 IRMS; Sercon) using air as the reference standard; the IRMS detector was regularly calibrated to commercially available reference gases. The ^15^N transferred from fungus to plant was then calculated using equations published by Field *et al*. (2016).

The ^14^C activity of shoot, root and fungal samples was quantified through sample oxidation (307 Packard Sample Oxidation, Isotech) followed by liquid scintillation. Total C (^12^C + ^14^C) fixed by the plant and transferred to fungus was calculated as a function of the total volume and CO_2_ content of the labelling chamber and the proportion of the supplied ^14^CO_2_ label fixed by plants. As severing the fungal hyphae only impacts the movement of ^33^P/^15^N from the well to the plant, the difference in total C between the values obtained for clover cultures with MFRE and those without fungus is considered equivalent to the total C transferred from plant to symbiotic fungus within the Phytagel for that microcosm, noting that a small proportion will be lost through respiration and accounting for plant‐fixed C gained by MFRE via diffusion, root exudation and/or dark fixation. The total C budget for each microcosm was calculated using equations adapted from Cameron *et al*. (2007) (Eqn 2). Total per cent allocation of plant‐fixed C to extraradical symbiotic fungal hyphae was calculated by subtracting the activity (in Becquerels) of clover cultures without fungus from that detected in monoxenic cultures with MFRE present, dividing this by the sum of activity detected in all components of each microcosm, then multiplying by 100.

(Eqn 2) $$M_{c}=\left(\left(\frac{A}{\mathrm{SAct}}\right)M^{14}C\right)+\left(P_{r}\times M{\mathrm{wt}}_{c}\right)$$

where *M*_c_, mass of carbon transferred from plant to fungus; *A*, radioactivity of the tissue sample (Bq); SAct, specific activity of the source (Bq Mol^−1^); *M* ^14^C, atomic mass of 14C; *P*_*r*_, proportion of the total ^14^C label supplied present in the tissue; $M{\mathrm{wt}}_{c}$, mass of C in the CO_2_ present in the labelling chamber (g) (from the ideal gas law; Eqn 3):

(Eqn 3) Mcd=McdPVcdRT∴mc=mcd×0.27292

where *m*_cd_, mass of CO_2_ (g); *M*_cd_, molecular mass of CO_2_ (44.01 g mol^−1^); P, total pressure (kPa); *V*_cd_, volume of CO_2_ in the chamber (0.003 m^3^); *R*, universal gas constant (J K^−1^ mol^−1^); *T*, absolute temperature (K); *m*_c_, mass of C in the CO_2_ present in the labelling chamber (g), where 0.27292 is the proportion of C in CO_2_ on a mass fraction basis.

### Data analyses

Isotope tracing data were analysed in Spss Statistics v.26 (IBM, Armonk, NY, USA). Data were tested for normality and homogeneity of variances using the Kolmogorov–Smirnov test for normality. Where assumptions for parametric tests were not met, data were transformed using log_10_. If assumptions for parametric tests were still not met, a nonparametric statistical test would be performed. The differences between plant assimilation of ^33^P and ^15^N were tested using Mann–Whitney *U* and Kruskal–Wallis analyses. Whiskers on box plots represent each of the data points (minimum to maximum) recorded during data collection.

## Results

### Molecular identification of the MFRE fungus

The molecular identification of the fungus colonising the roots of clover *in vitro* was confirmed as the same fungus introduced in our monoxenic microcosms following isolation from wild gametophytes and young sporophytes of *L. inundata*, all of which matched (99.69%) Endogonales sp. GenBank KJ952213 (Rimington *et al*., 2015).

### Axenic culture of plants and fungi and *in vitro* colonisation of clover by MFRE

Axenic MFRE isolates (Fig. 1a) comprised fine hyphal networks with small intercalary and terminal swellings < 20 μm in diameter (Fig. 1b,d). After *c*. 12 wk from the establishment of monoxenic microcosms containing axenic clover seedlings and MFRE hyphae, abundant mycelium extended from the original plugs of pure MFRE cultures (Fig. 1e) and enveloped the roots of clover (Fig. 1f). Root colonisation by MFRE was confirmed by molecular methods and staining (Fig. 1g–i), which revealed copious fungal colonisation with cytology typical of MFRE, consisting of fine (< 1.5 μm in diameter) irregularly branching hyphae with small intercalary and terminal swellings (Fig. 1g,h), sometimes forming tightly wound coils and hyphal ropes (Fig. 1h), spores or vesicles (Fig. 1g) and fine arbuscule‐like structures (Fig. 1i) in the root cortical cells of clover, with arbuscule‐like structures confined to the inner cortex.

### MFRE directly transfer ^15^N and ^33^P to clover

**Fig. 3:**
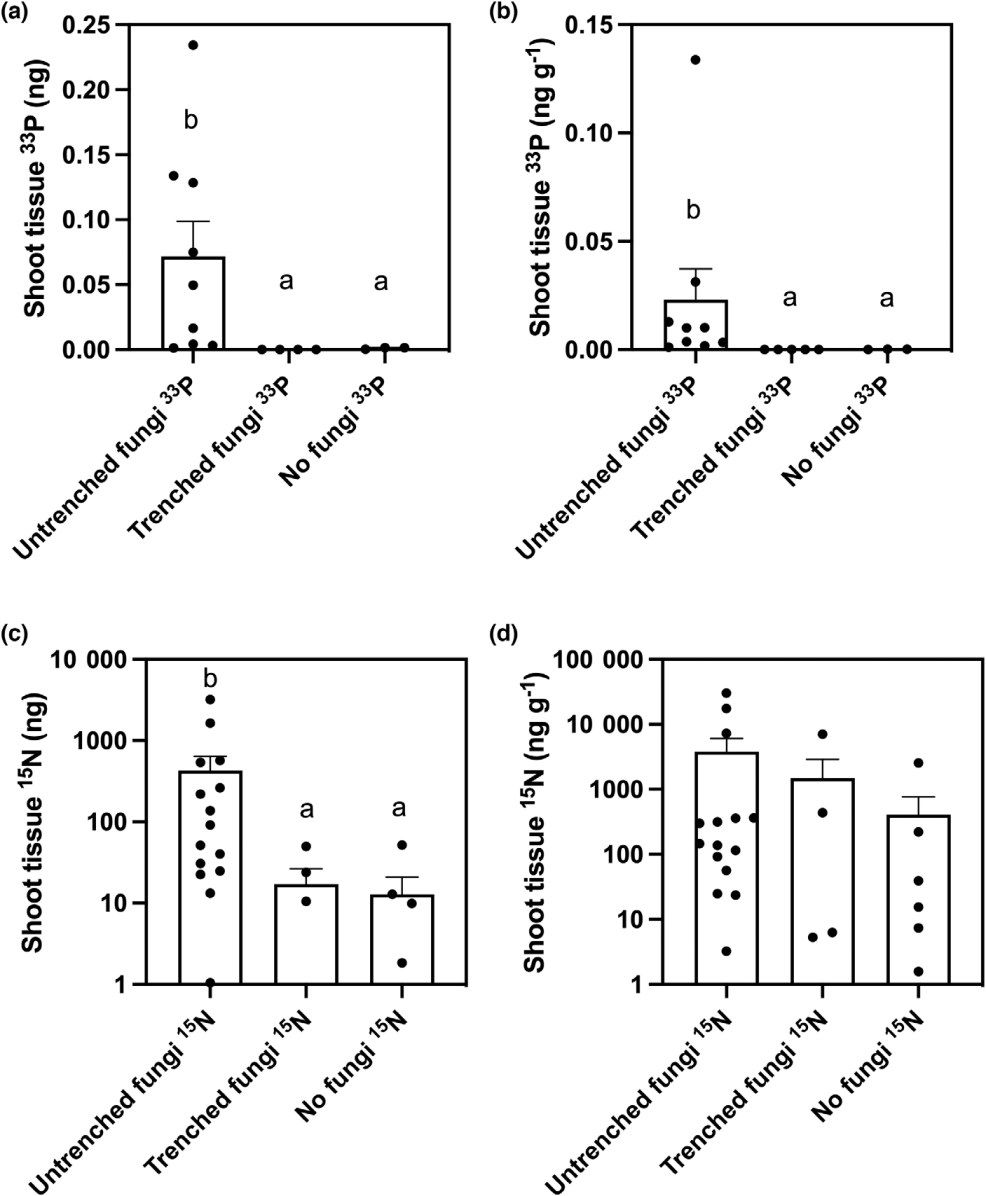
Nutrient fluxes between white clover (*Trifolium repens*) and Mucoromycotina fine root endophytes (MFRE). (a, b) Total shoot tissue phosphorus (^33^P) in nanograms (ng) in sterile monoxenic cultures with intact fungi, trenched fungi and with no fungi present (a) and shoot concentration (ng g^−1^) of fungal‐acquired ^33^P in *T. repens* (b). (c, d) Total shoot tissue (^15^N) in nanograms (ng) in sterile monoxenic cultures with intact MFRE fungi (c) and shoot concentrations (ng g^−1^) of fungal‐acquired ^15^N in *T. repens* (d). In (a, b), *n* = 9 for monoxenic cultures with intact fungi and *n* = 5 for those with trenched fungi and *n* = 3 for monoxenic cultures with no fungi present. In (c, d), *n* = 16 for monoxenic cultures with intact fungi and *n* = 5 for those with trenched fungi and *n* = 6 for monoxenic cultures with no fungi present (*n* indicates the number of biological replicates used during radio‐ and stable‐isotope tracing experiments). Letters denote significant differences where *P* < 0.05, Mann–Whitney *U* and Kruskal–Wallis tests. Error bars represent the standard error of the mean, with all data points shown (minimum to maximum) collected during the experiments.

In the experimental microcosms where fungal hyphae remained intact (Fig. 2a), MFRE transferred an average of 48% (±18%) of the supplied ^33^P tracer and an average of 43% (±21%) of the supplied ^15^N tracer to the shoots of clover during the labelling period. We determined significant transfer of fungal‐acquired ^33^P to clover by comparing the amount of ^33^P in shoot tissues where MFRE hyphae remained intact against amounts of ^33^P in shoot tissue in microcosms where hyphae were severed (Fig. 3a; *P* = 0.003; Table 1) or where MFRE was not present (Fig. 3a; *P* = 0.036; Table 1) in terms of absolute quantities and when normalised to plant biomass (Fig. 3b; *P* = 0.057; Table 1). There was significantly more ^15^N present in shoots where MFRE hyphae remained intact than in shoot tissue in microcosms where hyphae were severed (Fig. 3c; *P* = 0.04; Table 1) and axenic plates without MFRE (Fig. 3c; *P* = 0.01; Table 1) in terms of absolute quantities. However, when ^15^N assimilation was normalised to plant biomass, the transfer was not significant (Fig. 3d, severed hyphae, *P =* 0.349; no MFRE present, *P* = 0.056; Table 1).

**Table 1:** Summary of statistical results of ^15^N and ^33^P isotope tracing experiments.

		Intact fungi vs Trenched fungi	Intact fungi vs No fungi	Trenched fungi vs No fungi
Absolute values ^33^P (ng)				
Mann–Whitney *U P*‐value		0.003	0.036	0.057
Kruskal–Wallis *P‐*value	0.004			
Concentration values [^33^P] (ng g^−1^)				
Mann–Whitney *U P*‐value		0.001	0.009	0.036
Kruskal–Wallis *P‐*value	0.001			
Absolute values ^15^N (ng)				
Mann–Whitney *U P*‐value		0.040	0.010	0.662
Kruskal–Wallis *P‐*value	0.013			
Concentration values ^15^N (ng g^−1^)				
Mann–Whitney *U P*‐value		0.349	0.056	1.000
Kruskal–Wallis *P‐*value	0.156			

### Plant‐fixed carbon is detected in extraradical MFRE hyphae

**Fig. 4:**
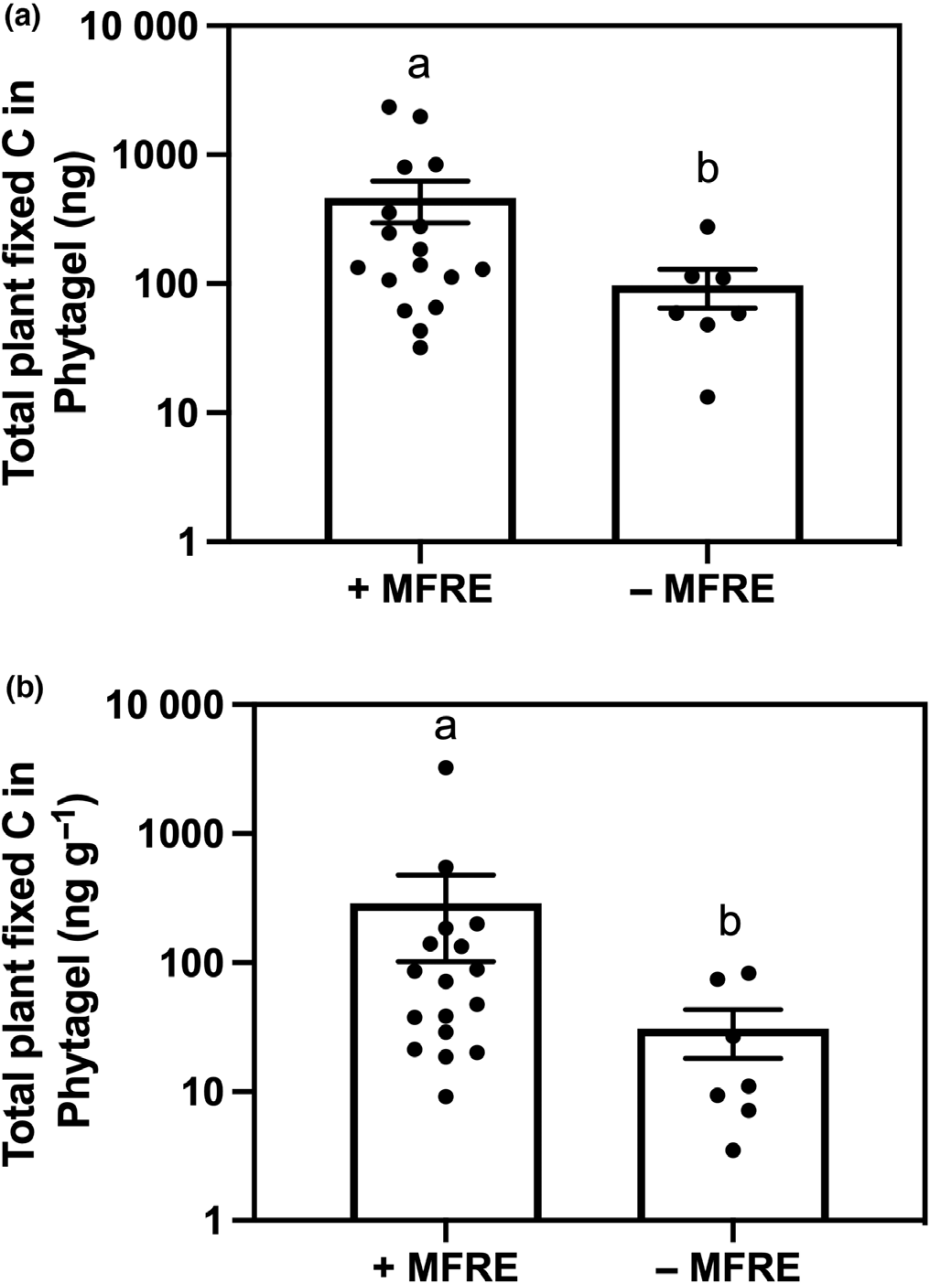
Total plant‐derived carbon present in extraradical Mucoromycotina fine root endophytes (MFRE) fungal hyphae. (a) Total plant‐derived carbon present in Phytagel with MFRE fungi present or plant‐only microcosms where no MFRE were present after a 24‐h labelling period (ng) and concentrations (ng g^−1^) (b). For both (a) and (b), *n* = 17 for microcosms with MFRE present and *n* = 7 for microcosms with no MFRE present. Letters denote significant differences where *P* < 0.05, Mann–Whitney *U* and Kruskal–Wallis tests. Error bars represent the standard error of the mean, with all data points shown (minimum to maximum) collected during the experiments.

Based on the drawdown and detection of ^14^C in plant and fungal materials over the 24‐h labelling period, we calculated a complete C budget for each microcosm (Supporting Information Fig. S1). There was significantly more plant‐fixed carbon present in microcosms containing MFRE than in plant‐only microcosms (Fig. 4a,b; *P* = 0.047). 606.29 ng (440.73 ng g^−1^) of plant‐fixed C was present in extraradical MFRE hyphae (Fig. 4a,b), equivalent to 0.87% of the total amount of C fixed by the clover during the 24‐h labelling period.

## Discussion

Our results provide the first unequivocal demonstration that symbiosis between a flowering plant, white clover, and a MFRE fungus is mutualistic, with the plant gaining both ^15^N and ^33^P tracers directly from the fungus while the fungus gains plant‐fixed C, in the absence of other micro‐organisms. While we focussed on a specific association, when considered together with those of previous studies (Field *et al*., 2015b, 2016, 2019; Orchard *et al*., 2017b; Hoysted *et al*., 2019, 2020; Albornoz *et al*., 2020, 2021), our findings indicate that MFRE symbioses are nutritionally mutualistic across diverse land plants.

Analysis of the fungus colonising the roots of clover, confirmed molecularly as an Endogonales (Mucoromycotina) isolate from a lycophyte (Hoysted *et al*., 2019, 2020), revealed a morphology characterised by fine, irregularly branching hyphae with small intercalary and terminal swellings (Fig. 1g,h), forming vesicles or spores (Fig. 1g) as well as hyphal coils and hyphal ropes (Fig. 1h) alongside arbuscule‐like structures (Fig. 1i). This morphology matches that described previously in a range of vascular (Orchard *et al*., 2017b; Hoysted *et al*., 2019, 2020; Albornoz *et al*., 2020) and nonvascular plants (Field *et al*., 2015b, 2016, 2019) colonised by MFRE.

To date, MFRE research has been carried out using unpasteurised soil culture‐based experimental systems (Orchard *et al*., 2017a; Albornoz *et al*., 2020) or wild‐collected plants (Field *et al*., 2015b, 2016, 2019; Hoysted *et al*., 2020). As such, it is inevitable that these experiments included other soil micro‐organisms alongside MFRE. As there is no information about how rhizosphere bacteria may influence MFRE metabolic characteristics and function, the inclusion of soil micro‐organisms in previous studies made it impossible to determine the direct contribution of MFRE to host plant nutrition. Here, we show for the first time, using a novel *in vitro* monoxenic system, that MFRE directly assimilate and transfer both ^33^P and ^15^N to a flowering plant in the absence of other microbes.

Our results reveal significant transfer of fungal‐acquired ^33^P to clover; however, while we observed a clear trend of greater [^15^N] in plant shoots when MFRE hyphae are intact than where they are severed, the difference is not statistically significant. A possible explanation is that clover, a legume, is not heavily reliant on fungal symbionts for N assimilation, even in the absence of rhizobia. This could also be due to revolatilisation and recapture of ammonium by the plant, and/or mass flow driven by plant transpiration in these microcosms. Nevertheless, and although the abundance of MFRE in our microcosms was not quantified, our results, when tentatively compared with those of previous studies on AMF (Thirkell *et al*., 2020a,b), indicate that clover (without rhizobia) may assimilate more ^15^N tracer via its MFRE symbiont per unit plant biomass than is typically assimilated by plants associated with AMF, albeit in nonsterile systems. This nutritional role, already indicated by studies of MFRE symbioses in nonflowering plants (Field *et al*., 2019; Hoysted *et al*., 2019), could help to explain the persistence of MFRE across most modern land plant lineages, facilitating plant access and assimilation of soil N (Howard *et al*., 2022). The FRE have long been thought to enhance plant P, at least in soils with very low plant‐available P (Crush, 1973; Rabatin *et al*., 1993; Orchard *et al*., 2017b; Albornoz *et al*., 2021); however, their potential role in plant N uptake has been overlooked. It is therefore important that N transfer and assimilation from MFRE to plant hosts are now investigated *in vitro* across a range of other flowering plants that do not associate with N‐fixing bacteria. Parallel studies of N and P transfer by AMF are also required before meaningful comparisons between AMF and MFRE function can be made.

Because we used an inorganic N source, additional experiments are also needed to assess potential direct organic N utilisation by MFRE (Field *et al*., 2019). A recent study on the ability of AMF to utilise N from organic sources (Rozmoš *et al*., 2022) showed, using an *in vitro* monoxenic experimental system based on Ri T‐DNA transformed chicory roots, that organic nitrogen utilisation by *Rhizophagus irregularis* was mediated by specific soil bacteria and accelerated by the presence of a protist. These findings, though not based on full plants, may explain the results of previous experiments using organic matter patches labelled with ^15^N in soil‐based microcosms, which showed successful transfer of the ^15^N by AMF to host plants (Hodge *et al*., 2000, 2001; Thirkell *et al*., 2016). Thus, it is likely that AMF‐associated and free‐living rhizospheric bacteria as well as other soil fungi contained in the nonsterile fungal inoculum used in those experiments may have interacted with AMF (Vivas *et al*., 2003; Frey‐Klett *et al*., 2007; Smith & Smith, 2011; Jiang *et al*., 2021), influencing the breakdown, mineralisation and assimilation of ^15^N‐labelled organic material. It is now important to determine whether similar processes may also explain organic N utilisation by MFRE (Field *et al*., 2019) or whether these fungi, by virtue of their putative facultatively saprotrophic nature, can directly access and transfer N from organic sources. Since organic N represents a large proportion of total soil N, direct organic N utilisation by MFRE would have important implications for terrestrial N cycling (Hodge & Storer, 2015; Howard *et al*., 2022).

Further research using monoxenic systems is also needed to compare the ‘cost’ in terms of plant‐to‐fungus transfer of C between AMF and MFRE symbioses. Our data show that symbiotic MFRE gain clover‐fixed C (Fig. 4a,b), and previous experiments using soil culture‐based systems suggested that the ‘cost’ of MFRE‐vascular plant associations is at least on a par with, if not larger than, AMF‐vascular plant associations (Hoysted *et al*., 2020). However, this has only been compared in one vascular plant species; whether it holds true for *in vitro* monoxenic systems remains to be tested.

Previous research into the function of plant–MFRE symbioses raised fundamental questions about their persistence and ecological relevance in modern terrestrial ecosystems. We can now begin to address such questions with new experimental systems knowing that symbiotic MFRE are nutritionally mutualistic with flowering plants. Field *et al*. (2015b) demonstrated the ability of MFRE isolates to recolonise host liverworts *in vitro*; however, until now, this had not been achieved in vascular plants. Our *in vitro* system used a fungal isolate that originated from a wild‐collected early‐diverging vascular plant, *L. inundata*, and was introduced to white clover *in vitro*. This isolate was molecularly and cytologically confirmed to be colonising the roots of clover used in our experiment. This represents a novel, tractable *in vitro* experimental system designed to manipulate MFRE isolates and the resynthesis of their mycorrhizas with a flowering plant. It opens a realm of exciting possibilities for further research on MFRE mycorrhizal properties, including cytological, molecular and metabolomic comparisons with AMF where host plants are inoculated singly or co‐colonised with both MFRE and AMF. Furthermore, a fundamental understanding of how MFRE distribution and function are affected by environmental factors such as temperature, water, light and atmospheric CO_2_, in addition to biotic factors such as interactions with other soil microbiota, can now be developed. Successful isolation from wild plants and axenic cultivation of an MFRE isolate offers exciting new opportunities to develop a model system for symbiotic MFRE and for omics in comparison with other fungi.

## Competing interests

None declared.

## Author contributions

KJF, SP, MIB and GAH conceived and designed the investigation. SP collected the plant material, carried out the isolation of fungal symbionts and designed the monoxenic cultures with the help of GAH. KJF and CAB undertook the isotope tracing. GAH led the data analysis and writing. BS designed Figure 2 and contributed to the writing. All authors discussed the results and commented on the article. SP and KJF agree to serve as the authors responsible for contact and ensure communication.

## Supporting information


**Fig. S1** Total carbon flux budget for monoxenic *in vitro* cultures of Mucoromycotina fine root endophyte hyphae (MFRE) colonising white clover (*Trifolium repens*).
Please note: Wiley is not responsible for the content or functionality of any Supporting Information supplied by the authors. Any queries (other than missing material) should be directed to the *New Phytologist* Central Office.

## Data Availability

The data generated in this study are provided in the Main Manuscript file. Full datasets are available from the corresponding authors upon reasonable request. Sequence data have been deposited in GenBank with accession no. KJ952213.
