# Supplementary material for: Direct nitrogen, phosphorus and carbon exchanges between Mucoromycotina ‘fine root endophyte’ fungi and a flowering plant in novel monoxenic cultures
Source: New Phytol. 2023 Feb 5;238(1):70–9. doi: 10.1111/nph.18630 (PMC10952891; doi:10.1111/nph.18630)
Supplement: Supplementary file 1 — Fig. S1 Total carbon flux budget for monoxenic in vitro cultures of Mucoromycotina fine root endophyte hyphae (MFRE) colonising white clover (Trifolium repens). Please note: Wiley is not responsible for the content or functionality of any Supporting Information supplied by the authors. Any queries (other than missing material) should be directed to the New Phytologist Central Office. [file NPH-238-70-s001.pdf]

New Phytologist Supporting Information

Article title: Direct nitrogen, phosphorus and carbon exchanges between Mucoromycotina ‘fine root endophyte’ fungi and a flowering plant in novel monoxenic cultures

Authors: Grace A. Hoysted, Katie J. Field, Besiana Sinanaj, Christopher A. Bell, Martin I. Bidartondo, Silvia Pressel

Article acceptance date: 15 November 2022

Fig. S1. Total carbon flux budget for monoxenic *in vitro* cultures of Mucoromycotina fine root endophyte hyphae (MFRE) colonising white clover (*Trifolium repens*). (a) Total plant-derived carbon present in Phytagel, shoots and roots with MFRE fungi present (white bars, +MFRE) or Phytagel, shoots and roots where no MFRE were present (grey bars, -MFRE) after a 24-hour labelling period (ng) and concentrations (ng g^{-1}) (b). For both (a) and (b) n = 17 for microcosms with MFRE present and n = 7 for microcosms with no MFRE present. Letters denote significant differences where $P < 0.05$, Mann-Whitney U and Kruskal-Wallis test. Error bars represent the standard error of the mean (SEM), with all data points shown (minimum to maximum) collected during the experiments.

A

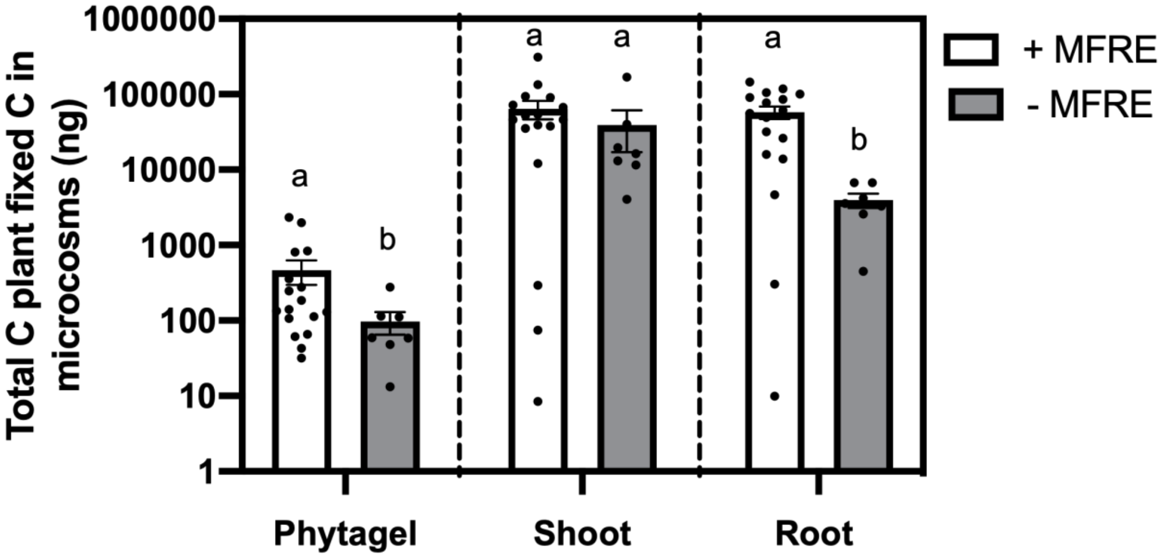

Total C plant fixed C in microcosms (ng)
1000000
100000
10000
1000
100
10
1
a
b
a
a
a
b
+ MFRE
- MFRE
Phytagel
Shoot
Root

B

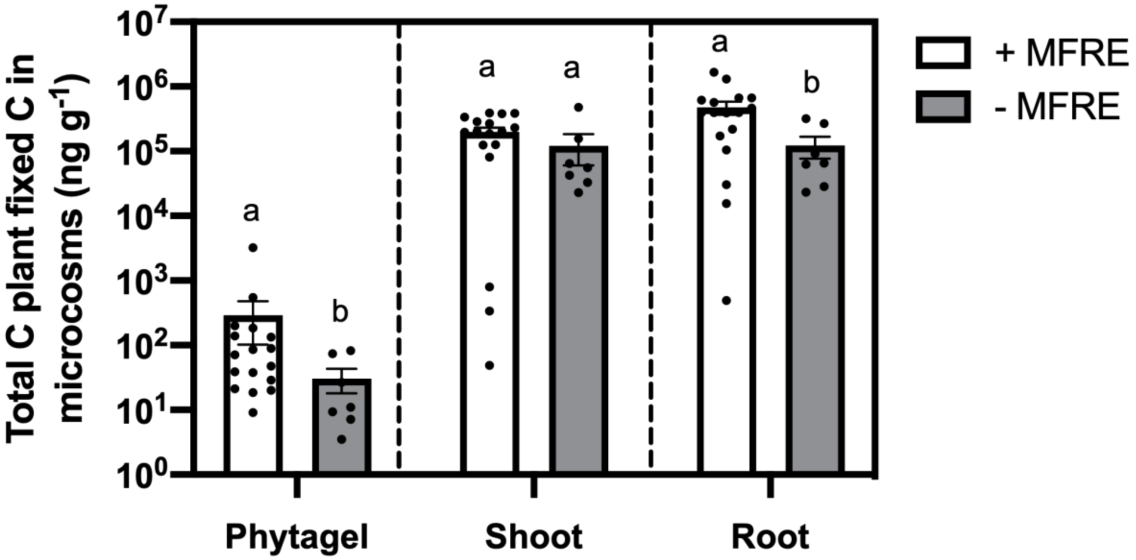

Total C plant fixed C in microcosms (ng g-1)
10^7
10^6
10^5
10^4
10^3
10^2
10^1
10^0
a
b
a
a
a
b
+ MFRE
- MFRE
Phytagel
Shoot
Root
